# Electrocardiographic markers of atrial cardiomyopathy and risk of heart failure in the multi-ethnic study of atherosclerosis (MESA) cohort

**DOI:** 10.3389/fcvm.2023.1143338

**Published:** 2023-04-26

**Authors:** Muhammad Imtiaz Ahmad, Mohammadtokir Mujtaba, James S. Floyd, Lin Y. Chen, Elsayed Z. Soliman

**Affiliations:** ^1^ Department of Internal Medicine, Section on Hospital Medicine, Medical College of Wisconsin, Wauwatosa, WI, United States; ^2^ Department of Internal Medicine, Section on Hospital Medicine, Geisel School of Medicine, Dartmouth, NH, United States; ^3^ Departments of Medicine and Epidemiology, University of Washington, Seattle, WA, United States; ^4^ Lillehei Heart Institute and Cardiovascular Division, University of Minnesota Medical School, Minneapolis, MN, United States; ^5^ Epidemiological Cardiology Research Center (EPICARE), Department of Epidemiology and Prevention, Wake Forest School of Medicine, Winston-Salem, NC, United States

**Keywords:** heart failure, atrial cardiomyopathy, electrocardiagram, prevention, MESA (Multi-Ethnic study of atherosclerosis)

## Abstract

**Background:**

The association of electrocardiographic (ECG) markers of atrial cardiomyopathy with heart failure (HF) and its subtypes is unclear.

**Methods:**

This analysis included 6,754 participants free of clinical cardiovascular disease (CVD), including atrial fibrillation (AF), from the Multi-Ethnic Study of Atherosclerosis. Five ECG markers of atrial cardiomyopathy (P-wave terminal force in V1 [PTFV1], deep-terminal negativity in V1 [DTNV1], P-wave duration [PWD], P-wave axis [PWA], advanced intra-atrial block [aIAB]) were derived from digitally recorded electrocardiograms. Incident HF events through 2018 were centrally adjudicated. An ejection fraction (EF) of 50% at the time of HF was used to classify HF as HF with reduced EF (HFrEF), HF with preserved EF (HFpEF), or unclassified HF. Cox proportional hazard models were used to examine the associations of markers of atrial cardiomyopathy with HF. The Lunn-McNeil method was used to compare the associations in HFrEF vs. HFpEF.

**Results:**

413 HF events occurred over a median follow-up of 16 years. In adjusted models, abnormal PTFV1 (HR (95%CI): 1.56(1.15–2.13), abnormal PWA (HR (95%CI):1.60(1.16–2.22), aIAB (HR (95%CI):2.62(1.47–4.69), DTNPV1 (HR (95%CI): 2.99(1.63–7.33), and abnormal PWD (HR (95%CI): 1.33(1.02–1.73), were associated with increased HF risk. These associations persisted after further adjustments for intercurrent AF events. No significant differences in the strength of association of each ECG predictor with HFrEF and HFpEF were noted.

**Conclusions:**

Atrial cardiomyopathy defined by ECG markers is associated with HF, with no differences in the strength of association between HFrEF and HFpEF. Markers of atrial Cardiomyopathy may help identify individuals at risk of developing HF.

## Introduction

According to data from US National Survey from 2015 to 2018, approximately 6 million Americans aged ≥20 years had heart failure (HF) (1), and it is projected that the prevalence of HF would increase by 46% from 2012 to 2030 (1). Significant delays in the identification of HF among Blacks, Hispanic individuals, and women can potentially delay the initiation of effective interventions to slow the progression of HF (2, 3), contributing to disparities in diagnosis and treatment. Therefore, noninvasive, and cost-effective markers should be explored for the early detection of HF in these populations (4, 5).

Prior studies have shown that left atrial (LA) dysfunction and remodeling are associated with and precede the onset of HF in otherwise asymptomatic participants (6–8). In one study, approximately 45% of patients presenting with new-onset HF with preserved ejection fraction (HFpEF) had LA dysfunction as the underlying mechanism (9). Despite accumulating evidence suggesting that impaired LA function is an important pathogenic factor in the development of HF, especially HFpEF, there is no consensus about the optimal method for detecting LA dysfunction, which may ultimately prove to be a therapeutic target for the prevention of HF and other cardiovascular diseases (CVD). Several P-wave indices (PWIs), easily measured electrocardiographic (ECG) markers of atrial cardiomyopathy, have been associated with an increased risk of poor outcomes such as atrial fibrillation (AF), mortality, vascular injury, and stroke (10). However, no prior studies have systematically explored the association of ECG markers of atrial cardiomyopathy with incident HF in a population free of HF and atrial fibrillation (AF). We proposed to examine the association between several ECG markers of atrial cardiomyopathy and incident HF in MESA, a multiethnic prospective cohort study with over 16 years of follow-up adjudicated CVD events.

## Methods

### Study population

Between July 2000 and September 2002, 6,814 participants aged 45–84 years old were recruited at 6 field centers (Baltimore, Maryland; Chicago, Illinois; Forsyth County, North Carolina; Los Angeles, California; New York, New York; and St. Paul, Minnesota) (11). All participants were free of clinical CVD, including HF, at baseline. All participants provided informed consent, and the study protocol was approved by the institutional review board at each participating institution. For this analysis, participants were excluded if they were missing baseline ECG data, prior history of AF, or HF follow-up data.

### Baseline characteristics

Baseline covariates were recorded at the initial MESA examination. Age, gender, race/ethnicity, annual household income, education, and smoking history were self-reported. For this analysis, annual income and education were used as a categorical variable dichotomized at <$20,000 vs. ≥$20,000, and education was dichotomized at high school or less vs. college or higher education. Current smokers and former smokers were defined as ever-smokers vs. never-smokers. After a 12-hour fast, blood samples were drawn for total cholesterol, high-density lipoprotein cholesterol, and plasma glucose measurements. Low-density lipoprotein cholesterol (LDL-C) was calculated using the Friedewald equation: “*LDLc = total cholesterol−HDLc-triglycerides × 0.2*” (12). Diabetes mellitus was defined as a self-report history of diabetes or fasting glucose ≥126 mg/dl or the use of medications for diabetes. Antihypertensive medications, lipid-lowering medication, antidiabetic medications, and aspirin use were determined after the review of the medication containers brought to the clinic for verification. Systolic Blood pressure and diastolic blood pressure were measured using an automated sphygmomanometer with each participant seated for at least 5 min. Hypertension was defined as systolic blood pressure of ≥130 or diastolic blood pressure of ≥80 or the use of antihypertensive medications (13). Body mass index was reported as the weight in kilograms divided by the square of the height in meters. The estimated glomerular filtration rate (eGFR) was calculated by the chronic kidney disease epidemiology (CKD-EPI) collaboration formula (14).

### ECG measurements

Using standardized procedures, 12-lead ECGs were obtained by trained technicians using GE MAC 1,200 electrocardiographs. After electronic transmission, ECGs were ready at the ECG Reading Center at the Epidemiological Cardiology Research Center (Wake Forest School of Medicine, Winston-Salem, NC). After visual inspection for errors or poor quality, all ECGs were automatically processed using the 2001 version of the GE Marquette 12-SL program. P-wave areas, amplitudes, and durations were automatically measured using the same program (15). P-wave terminal force in V1 (PTFV_1_) was calculated using the duration of the downward deflection terminal portion of the P-wave in lead V_1_ in milliseconds (ms) multiplied by the absolute value of its amplitude in microvolts (µV). Abnormal PTFV1 was defined as values >5,000 µV × ms (16). The deep terminal negative phase of the P-wave in lead V1 (DTNPV1) values ≥100 µV were considered abnormal. Any value outside the range of 0° and 75° defined abnormal P-wave axis (aPWA). Abnormal P-wave duration (PWD) was defined as values >120 ms. Advanced IAB (aIAB) was reported as the presence of P-wave duration ≥120 ms and biphasic (positive-negative) morphology in leads II, III, and aVF. Supplementary Figure S1 demonstrates these abnormal P-wave indices.

### Heart failure

MESA participants or their next of kin were contacted by trained staff every 9–12 months following MESA enrollment to inquire about any hospitalizations and if any hospitalization, medical records for those hospitalizations were reviewed for hospitalized HF events. The review and diagnosis of hospitalized HF events were adjudicated by a panel of physician review committee using standardized criteria. We included probable and definite hospitalized HF events in this analysis. Probable HF was ascertained by a previous physician's diagnosis of HF or treatment for HF. Definite HF required confirmed hospitalization with heart failure and at least one additional objective criterion such as pulmonary edema or congestion by chest radiography, dilated ventricle, or reduced left ventricular function by echocardiography or ventriculography, or evidence of left ventricular diastolic dysfunction. Participants were followed from the baseline visit until newly diagnosed HF, death, drop-out, or until December 31, 2018. HF events were classified by EF and reported in the medical records at the time of hospitalization. As there were few HF events in mid-range EF category (40%–49%), we used the cut-off of EF 50% to classify HF as HFpEF if EF ≥50% or HFrEF if EF <50% to consider two categories of HF subtypes.

### Atrial fibrillation

Incident AF during follow-up was identified from a combination of follow-up study ECGs, hospital discharge diagnosis of AF, and, from inpatient, outpatient, and physician AF claim data for participants enrolled in fee-for-service-Medicare. Any claims data for before to baseline MESA examination was determined to be prevalent AF and was excluded from the current analytical plan.

### Statistical analysis

Baseline characteristics were compared by each ECG predictor. Categorical variables were reported as frequency and percentage, whereas continuous variables were reported as mean ± SD.

Incidence rates for overall HF and subtype of HF were calculated for each ECG marker and were reported as 1,000-person years. Assumptions for Cox proportional hazard model were tested. Cox regression analysis was used to compute hazard ratios (HRs) and 95% confidence intervals for the association of each ECG marker with HF. Similarly, separate analyses were conducted to examine the association of each ECG predictor with HFpEF and HFrEF. Multivariable models were adjusted as follows: model 1 adjusted for age, sex, race/ethnicity, income, and education; model 2 adjusted for model 1 covariates plus hypertension, smoking, diabetes mellitus, body mass index, LDL-C, aspirin, lipid-lowering agents, and eGFR; model 3 adjusted for model 2 plus AF a as time-varying covariate.

To examine potential heterogeneity, we explored the association of each ECG predictor with HF across subgroups stratified by age, sex, and race status adjusting for covariates as mentioned above.

We used the Lunn–McNeil method to test whether ECG predictors were associated with differential risk for HFrEF vs. HFpEF. HF Survival probabilities for each ECG predictor vs. control were compared using Kaplan-Meier's method, and significance was tested using log-rank test.

SAS version 9.4 (SAS Institute Inc, Cary, NC) and Stata version 17 were used for all data analyses. Two-sided *P* < 0.05 indicated statistical significance.

## Result

6,754 participants (mean age 62 ± 10 years, 53% women, 38% white, 12% Chinese American, 28% black, 22% Hispanic) were included in the final analysis. Baseline characteristics stratified by each ECG predictor are shown in Table 1. As shown, participants with abnormal ECG predictors were more likely to be older, men, Black individuals with the higher prevalence of CVD risk factors such as higher BMI, higher blood pressure, lower eGFR, and higher fasting glucose, who were more likely to use aspirin, antihypertensive medications, and anti-diabetic medications. AF developed more frequently among those with abnormal ECG predictors than those without it (Table 1).

**Table 1:** Baseline characteristics of the study participants.

	PTFV1		P-Wave Axis		aIAB		DTNPV1		PWD	
	Normal (*n* = 6,288)	Abnormal (*n* = 466)	Normal (*n* = 6,148)	Abnormal (*n* = 567)	Absent (*n* = 6,680)	Present (*n* = 56)	Absent (*n* = 6,679)	Present (*n* = 57)	Normal (*n* = 5,981)	Abnormal (*n* = 755)
Age (Years)	61.8 ± 10.2	66.4 ± 9.4*	61.9 ± 10.1	64.5 ± 10.5*	62.0 ± 10.2	70.0 ± 7.1*	62.1 ± 10.2	67.4 ± 9.3*	61.6 ± 10.1	66.3 ± 9.6*
Men	2,948 (46.8%)	238 (51%)*	2,875 (46.7%)	287 (50.6%)*	3,143 (47.0%)	37 (66.0%)*	3,148 (47.1%)	32 (56.1%)*	285 (44.8%)	495 (65.5%)*
Race										
*White*	2,451 (38.9%)	137 (29%)*	2,320 (37.7%)	249 (43.9%)*	2,557 (38.2%)	23 (41.0%)*	2,562 (38.3%)	18 (31.5%)*	2,291 (38.3%)	289 (38.2%)
*Chinese*	758 (12.0%)	46 (9.8%)	718 (11.6%)	81 (14.2%)	797 (11.9%)	3 (5.3%)	791 (11.8%)	9 (15.7%)	752 (12.5%)	48 (6.3%)
*Black*	1,661 (26.5%)	214 (45.9%)	1,717 (27.9%)	147 (25.9%)	1,849 (27.6%)	21 (37.5%)	1,842 (27.5%)	28 (49.1%)	1,603 (26.8%)	267 (35.3%)
*Hispanic*	1,418 (22.5%)	69 (14.8%)	1,393 (22.6%)	90 (15.8%)	1,477 (22.1%)	9 (16.0%)	1,484 (22.2%)	2 (3.5%)	1,335 (22.3%)	151 (20.0%)
Income <20,000	1,673 (26.6%)	143 (31%)*	1,629 (26.5%)	182 (32.1%)*	1,800 (26.9%)	14 (25.0%)	1,793 (26.8%)	21 (36.8%)*	1,608 (26.8%)	206 (27.2%)
High School or Less	2,298 (36.5%)	162 (34.7%)	2,241 (36.4%)	210 (37.0%)	2,432 (36.4%)	25 (44.6%)*	2,433 (36.4%)	24 (42.1%)*	2,186 (36.5%)	271 (35.8%)
BMI	28.2 ± 5.4	29.4 ± 6.2*	28.5 ± 5.4	25.7 ± 5.3*	28.3 ± 5.4	29.7 ± 5.0*	28.3 ± 5.4	27.8 ± 5.1*	28.1 ± 5.4	29.4 ± 5.4*
SBP (mm Hg)	126.0 ± 21.2	135.8 ± 23.3*	126.7 ± 21.4	125.7 ± 22.5*	126.6 ± 21.4	135.1 ± 19.1*	126.6 ± 21.4	134.3 ± 22.9*	126.1 ± 21.2	131.0 ± 22.9*
DBP (mm Hg)	71.8 ± 10.2	73.8 ± 11.0*	72.0 ± 10.2	70.4 ± 10.5*	71.9 ± 10.2	74.6 ± 9.8*	71.9 ± 10.2	73.6 ± 11.7*	71.7 ± 10.2	73.3 ± 10.5*
LDL-C (mg/dl)	117.5 ± 31.5	114.4 ± 30.8*	117.4 ± 31.4	115.9 ± 32.1*	117.3 ± 31.4	107.7 ± 31.0*	117.3 ± 31.5	109.7 ± 30.5*	117.6 ± 31.6	114.1 ± 30.1*
Glucose (mg/dl)	96.9 ± 29.7	103.5 ± 36.6*	97.8 ± 30.9	92.1 ± 22.8*	97.3 ± 30.3	98.8 ± 19.5*	97.3 ± 30.3	98.7 ± 24.0*	96.9 ± 30.3	100.8 ± 29.8*
Smoking Status										
*Never*	3,177 (50.6%)	212 (46%)*	3,129 (51.0%)	240 (42.4%)*	3,346 (50.2%)	31 (55.3%)*	3,355 (50.3%)	22 (38.6%)*	3,026 (50.7%)	351 (46.6%)*
*Former*	2,285 (36.4%)	180 (38.8%)	2,213 (36.1%)	235 (41.5%)	2,437 (36.5%)	23 (41.0%)	2,434 (36.5%)	26 (45.6%)	2,129 (35.6%)	331 (44.0%)
*Current*	810 (12.9%)	71 (15.3%)	788 (12.8%)	91 (16.0%)	878 (13.1%)	2 (3.5%)	871 (13.0%)	9 (15.7%)	810 (13.5%)	70 (9.3%)
Antihypertensives	2,268 (36.0%)	245 (52%)*	2,297 (37.3%)	197 (34.7%)*	2,471 (37.0%)	34 (60.7%)*	2,472 (37.0%)	33 (57.8%)*	2,104 (35.2%)	401 (53.1%)*
Lipid Lowering Agents	1,004 (16.0%)	83 (17.8%)	996 (16.2%)	84 (14.8%)*	1,077 (16.1%)	9 (16.0%)	1,078 (16.1%)	8 (14.0%)*	961 (16.1%)	125 (16.5%)
Aspirin Use	1,950 (31.0%)	171 (37%)*	1,945 (31.6%)	166 (29.2%)*	2,094 (31.3%)	24 (42.8%)*	2,096 (31.4%)	22 (38.6%)*	1,817 (30.3%)	301 (39.9%)*
Antidiabetics	587 (9.3%)	68 (14.5%)*	620 (10.0%)	33 (5.8%)*	649 (9.7%)	6 (10.7%)	647 (9.6%)	8 (14.0%)*	559 (9.3%)	96 (12.7%)*
eGFR	77.8 ± 16.2	75.7 ± 16.6*	77.9 ± 16.2	75.1 ± 15.8*	77.8 ± 16.2	70.5 ± 15.0*	77.7 ± 16.2	77.3 ± 17.7	78.0 ± 16.1	75.0 ± 17.0*
Developed AFib	874 (14.0%)	108 (23.%)*	854 (14.0%)	120 (21.7%)*	957 (14.4%)	22 (40.0%)*	966 (14.6%)	13 (22.8%)*	795 (13.4%)	184 (24.6%)*

PTFV1; p-terminal force in V1; eGFR, estimated glomerular filtration rate; DTNPV1, deep negativity of p-wave in V1, aIAB, advanced interatrial block; PWD, p-wave duration; SBP, systolic blood pressure; DBP, diastolic blood pressure; AFib, Atrial Fibrillation.

* Represent variables across each ECG predictor with p-value < .0.05.

Continuous variables are presented as mean (standard deviation) and categorical variables as count (percentage).

Over a median follow-up of 16.6 years, a total of 413 HF cases (incidence rate of 4.34 per 1,000 person-years) were identified. Among those, there were 197 cases of HFrEF, 149 cases of HFpEF, and re unclassified cases of HF.

**Figure 1:**
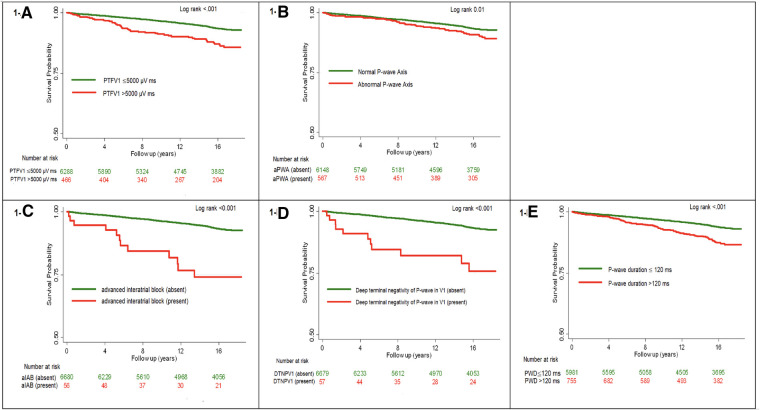
Unadjusted kaplan-meier survival curves for all incident heart failure stratified by each ECG predictor.

Figure 1 shows the unadjusted Kaplan-Meier survival probability curves stratified by each ECG predictor. Survival analysis revealed higher incidence of HF with each ECG predictor compared with the control (log-rank p-value <0.001). Supplementary Figure S2 shows covariate-adjusted Kaplan-Meier survival curves.

Results of HRs for total HF, HFrEF, and HFpEF are shown in Table 2. As shown, abnormal PTFV1 (>5,000 µV ms vs. ≤5,000 µV ms), and each standard deviation (SD) (1,832 µV ms) increase in PTFV1 were significantly associated with total HF in a model adjusted for demographics, CVD risk factors, and incident AF. Similarly, other ECG markers, DTNPV1, aIAB, aPWA and PWD were also significantly associated with the total HF in fully adjusted models and the associations persisted even after further adjustment for incident AF. No significant differences the in strength of the association of ECG predictors with the type of HF (HFrEF vs. HFpEF) were noted (Table 2). The proportional hazard assumptions were not violated.

**Table 2:** Association of atrial cardiomyopathy with heart failure.

Atrial Cardiomyopathy Markers	HF Group*	Events/1,000 PY	Model 1	Model 2	Model 3	*χ*2 *P* For HF Subtype Difference!
			HR (95% CI)	HR (95% CI)	HR (95% CI)	
P-terminal Force in V1 (PTFV1) (PTFV1 > 5,000 µv.ms)	All HF	8.92	**1.80 (1.32–2.43)**	**1.56 (1.15–2.13)**	**1.55 (1.14–2.12)**	
	HFrEF	3.39	1.38 (0.85–2.26)	1.25 (0.76–2.05)	1.18 (0.72–1.94)	0.09
	HFpEF	3.47	**2.32 (1.45–3.70)**	**2.03 (1.27–3.26)**	**2.12 (1.32–3.41)**	
Each SD increase in PTFV1 (1 SD = 1,832 µV ms)	All HF	4.34	**1.24 (1.14–1.36)**	**1.19 (1.08–1.30)**	**1.17 (1.07–1.29)**	
	HFrEF	2.07	**1.22 (1.07–1.39)**	**1.17 (1.02–1.34)**	**1.13 (0.99–1.30)**	0.21
	HFpEF	1.56	**1.33 (1.16–1.54)**	**1.26 (1.10–1.45)**	**1.28 (1.11–1.48)**	
Abnormal P-wave axis (aPWA) (P-wave axis outside the range of 0–75°)	All HF	6.08	1.27 (0.93–1.75)	1.61 (1.17–2.22)	1.40 (1.01–1.94)	
	HFrEF	2.00	0.88 (0.52–1.51)	1.08 (0.63–1.84)	0.96 (0.56–1.64)	0.34
	HFpEF	2.25	1.31 (0.78–2.22)	1.65 (0.97–2.81)	1.39 (0.80–2.40)	
Advanced Intra-atrial block (aIAB)	All HF	19.1	**2.77 (1.55–4.95)**	**2.62 (1.47–4.69)**	**2.55 (1.42–4.57)**	
	HFrEF	12.7	**4.20 (2.05–8.61)**	**3.99 (1.94–8.19)**	**3.79 (1.84–7.80)**	0.08
	HFpEF	1.59	0.65 (0.09–4.66)	0.60 (0.08–4.33)	0.59 (0.08–4.26)	
Deep terminal negativity-V1 (DTNPV1) (DTNPV1 ≥ 100 µv)	All HF	17.9	**3.30 (1.80–6.02)**	**3.08 (1.68–5.64)**	**3.45 (1.88–6.32)**	
	HFrEF	8.16	**3.22 (1.32–7.86)**	**2.99 (1.22–7.33)**	**3.10 (1.26–7.60)**	0.50
	HFpEF	8.16	**4.34 (1.77–10.6)**	**3.87 (1.57–9.54)**	**4.78 (1.93–11.7)**	
Prolonged P-wave duration (PWD) (P-dur >120 ms)	All HF	8.03	**1.43 (1.10–1.86)**	**1.33 (1.02–1.73)**	**1.30 (1.01–1.69)**	
	HFrEF	3.70	1.37 (0.94–2.01)	1.32 (0.90–1.93)	1.27 (0.86–1.86)	0.87
	HFpEF	2.67	1.45 (0.93–2.26)	1.31 (0.84–2.04)	1.33 (0.85–2.09)	
Each SD increase in P-wave duration (1 SD = 13.3 ms)	All HF	4.36	**1.18 (1.06–1.31)**	**1.12 (1.01–1.24)**	**1.12 (1.00–1.25)**	
	HFrEF	2.08	**1.20 (1.03–1.39)**	**1.16 (1.00–1.35)**	**1.13 (0.97–1.32)**	0.88
	HFpEF	1.57	1.16 (0.97–1.38)	1.09 (0.91–1.30)	1.11 (0.92–1.33)	

CI, confidence interval; HF, heart failure; HFpEF, heart failure with preserved ejection fraction; HFrEF, heart failure with reduced ejection fraction.

Statistically significant results at P < 0.05 are in bold font.

*Model 1:* age, sex, race, income, education*. Model 2:* Model 1 covariates + smoking status, hypertension, diabetes mellitus, body mass index (continuous), LDL cholesterol, aspirin use, lipid-lowering agents, eGFR*. Model 3:* Model 2 plus time-varying incident atrial fibrillation.

* The entire sample, including non-cases and HFrEF, HFpEF, and unclassified events, was included in the analysis for “all HF”. !*P*-value comparing survival functions of HFrEF vs. HFpEF using Lunn-McNeil analysis in augmented data set; unclassified cases were excluded from the analytic sample in this analysis.

In subgroup analyses by age, sex, and race, the association of abnormal ECG predictors with total HF was homogenous among these subgroups (Supplementary Table S1).

## Discussion

The major findings of this analysis of data from the multiethnic cohort are the following; Firstly, we found that all ECG markers of atrial cardiomyopathy are associated with incident HF independent of traditional CVD risk factors. Secondly, these associations remained significant even after adjustment for incident AF. Thirdly, there was no heterogeneity in the n association of these markers with HF by age, sex, or race subgroups. Lastly, there were no differences in the association of ECG predictors with HFrEF vs. HFpEF.

LA structural and functional remodeling is associated with CVD and poor outcomes in participants with prior CVD (17). Although atrial function and remodeling are strongly influenced by LV hemodynamics, accumulating evidence suggests that atrial remodeling precedes and independently contributes to the systemic dysregulation associated with HF (6, 18). One of the pathophysiological mechanisms involved in driving and propagation of AF and HF is atrial cardiomyopathy, a condition defined as “any complex of structural, architectural, contractile, or electrophysiological changes affecting the atria with the potential to produce clinically relevant manifestations” (6, 19, 20). AF and HF, in turn, further worsen the atrial cardiomyopathy thus creating an uninterrupted cycle of disease progression. For example, there was a progressive decline in LA mechanics with a higher AF burden, which in turn, predicted worsening HF and progression of paroxysmal AF to persistent or permanent AF (21). Therefore, early recognition of subclinical atrial disease/atrial cardiomyopathy by any modality such as ECG, cardiac imaging, or biomarkers offers a window of opportunity for lifestyle changes, risk factor control, and potential antifibrotic therapies to prevent the progression to overt clinical atrial disease (22). Currently, a universal criterion for diagnosis of atrial cardiomyopathy is lacking, however, PWIs used in the current study are well-known markers of atrial remodeling/atrial cardiomyopathy and predicted risk of stroke independent of AF (23–26). Our findings of the association of these ECG markers with HF independent of AF in a multiethnic cohort free of CVD suggest the need to use these markers for risk stratification in high-risk populations with existing cardiometabolic risk factors.

Abnormal PTFV1 is an independent predictor of AF, ischemic/non-lacunar stroke, sudden cardiac death (SCD), dementia, and mortality (27). In addition, a study led by Liu et al. found a statistically significant association between abnormal PTFV1 and cardiac death or hospitalization for HF in patients with prior myocardial infarction (MI) (28). Abnormal PTFV1 was also associated with worse left ventricular (LV) diastolic function (29, 30), and predicted abnormal diastolic function in participants with hypertension and preserved EF (30) Several potential mechanisms are related to the development and linking of abnormal PTFV1 with poor outcomes through the LA's structural, functional, and electrical remodeling. The presence of abnormal PTFV1 predicts LA size (31, 32), the larger LA size was associated with more deep terminal negativity of the P-wave in lead V1, and thus abnormal PTFV1 (24). LA size is a known predictor of morbidity and mortality including new-onset AF, stroke, and death (17, 33–34). PTFV1 is also a predictor of LA function. There is a significant association between deep negative P-wave in V1 and minimum LA volume index, global LAEF, and LA reservoir function (24, 35). Weak LA strain was associated with terminal negativity of P-wave in lead V_1_ in dose resdose-response (24). In addition, diffuse LV fibrosis was independently associated with abnormal PTFV1, and LV fibrosis (potentially through LA fibrosis) slowed the interatrial conduction resulting in gradual prolongation of P-wave and negative P-wave in V1 (24). Taken together, these observations suggest abnormal PTFV1 derived from ubiquitously available ECG is a marker of atrial remodeling and LA dysfunction and suggests a potential predictive value of PTFV1 in HF risk assessment.

Although PTFV1 is a widely used ECG marker of atrial cardiomyopathy (16, 36), DTNPV1 has shown its value as a relatively good substitute for PTFV1 and a predictor of poor outcomes (37, 38). In all participant with the terminal negative portion of the P wave in V1 amplitude, ≥100 µV in-depth, the duration of that terminal negative P wave in the V1 was greater than 0.04 s (39). Similar to PTFV1, DTNPV1 is a marker of electrical and functional remodeling of the LA (24) and demonstrated a statistically significant prediction of the combined outcomes (CHF, AF events, fatal, or non-fatal CHD, or stroke) in the Atherosclerosis Risk in Communities (ARIC) study (39). An association of DTNPV1 with incident HF in our study further supports its potential utility in risk prediction in the general population. Unlike PTFV1, DTNPV1 can be visualized without requiring any calculations.

We also observed an association between aIAB and incident HF in our study. aIAB is a risk factor for AF, stroke, cognitive impairment, and mortality (40–42). Advanced age, hypertension, and coronary artery disease (CAD) are risk factors for aIAB development and are pathophysiologically linked to fibrotic atrial cardiomyopathy (FAC) and reduced LA strain indexes (42, 43). Thus, aIAB is associated with perturbations in the electromechanical function and therefore potentially represents another marker for HF prediction in the general population (44). The prevalence of aIAB in our sample is consistent with the overall low prevalence of aIAB of 0.1%–0.5% of the general adult population (41, 42).

Like other PWIs, aPWA, and PWD are also markers of atrial remodeling (32, 45–47) and therefore, likely explain the association of these markers with incident HF. Unlike other PWIs, P-wave axis is reported on contemporary ECG print outs and therefore, any abnormality in P-wave axis is easily recognizable, with the potential utility to identify high-risk populations.

### Strengths and limitations

The current study should be interpreted in the context of several limitations. Although several covariates were included in the models, residual confounding remains a concern. Due to a limited number of HF events, analysis for differences for subtype of HF may have been underpowered to observe clinically meaningful differences. We did not use cardiac imaging to assess LA size or functions, especially cardiac magnetic resonance imaging (CMR) which is the most sensitive and specific method for the detection in LA abnormalities. However, the use of CMR for risk stratification in the general population is not cost effective. In addition, P-wave measurements likely represent conduction abnormalities, filling pressures or possible underlying LA fibrosis which may not by accurately assessed by other modalities (48). We may have missed cases of subclinical AF that can potentially mediate the association of PWIs with incident HF and thus, create a bias away from null hypothesis. Finally, for this analysis, we only used PWIs measured at baseline examination, therefore unable to assess any longitudinal changes in the ECG intervals and their impact on the outcomes. The strength of our study includes a prospective study with 16 years of follow-up in asymptomatic individuals from a racially diverse population with the availability of comprehensive and standardized clinical data. In addition, comprehensive adjudication of CVD events by expert physicians and use of PWIs from digital ECGs which were read at central reading center further represent strengths of the current study.

## Conclusions

In conclusion, atrial cardiomyopathy defined by ECG is associated with incident HF. Our findings provide a rationale for utilizing these well-established markers of atrial cardiomyopathy to replicate these findings in other large independent cohorts and to assess whether these markers can be used for HF risk prediction.

## Data Availability

The original contributions presented in the study are included in the article, further inquiries can be directed to the corresponding author.

## References

[1] Tsao CW Aday AW Almarzooq ZI Alonso A Beaton AZ Bittencourt MS Heart disease and stroke statistics-2022 update: a report from the American heart association. Circulation. (2022) 145:e153–639. 10.1161/CIR.0000000000001052 35078371

[2] Sandhu AT Tisdale RL Rodriguez F Stafford RS Maron DJ Hernandez-Boussard T Disparity in the setting of incident heart failure diagnosis. Circulation: Heart Failure. (2021) 14:e008538. 10.1161/CIRCHEARTFAILURE.121.008538 34311559 PMC9070116

[3] Mehta H Armstrong A Swett K Shah SJ Allison MA Hurwitz B Burden of systolic and diastolic left ventricular dysfunction among hispanics in the United States: insights from the echocardiographic study of latinos. Circulation Heart Failure. (2016) 9:e002733. 10.1161/CIRCHEARTFAILURE.115.002733 27048764 PMC4826756

[4] Paulus WJ. H(2)FPEF score: at last, a properly validated diagnostic algorithm for heart failure with preserved ejection fraction. Circulation. (2018) 138:871–3. 10.1161/CIRCULATIONAHA.118.035711 30354456

[5] Segar MW Vaduganathan M Patel KV McGuire DK Butler J Fonarow GC Machine learning to predict the risk of incident heart failure hospitalization among patients with diabetes: the WATCH-DM risk score. Diabetes Care. (2019) 42:2298–306. 10.2337/dc19-0587 31519694 PMC7364669

[6] Habibi M Chahal H Opdahl A Gjesdal O Helle-Valle TM Heckbert SR Association of CMR-measured LA function with heart failure development: results from the MESA study. JACC Cardiovasc Imaging. (2014) 7:570–9. 10.1016/j.jcmg.2014.01.016 24813967 PMC4129378

[7] Guichard J-B Nattel S. Atrial cardiomyopathy. J Am Coll Cardiol. (2017) 70:756–65. 10.1016/j.jacc.2017.06.033 28774383

[8] Bisbal F Baranchuk A Braunwald E de Luna A B Bayés-Genís A. Atrial failure as a clinical entity: JACC review topic of the week. J Am Coll Cardiol. (2020) 75:222–32. 10.1016/j.jacc.2019.11.013 31948652

[9] Sanchis L Gabrielli L Andrea R Falces C Duchateau N Perez-Villa F Left atrial dysfunction relates to symptom onset in patients with heart failure and preserved left ventricular ejection fraction. Eur Heart J Cardiovasc Imaging. (2015) 16:62–7. 10.1093/ehjci/jeu165 25187609

[10] Chen LY Ribeiro ALP Platonov PG Cygankiewicz I Soliman EZ Gorenek B P wave parameters and indices: a critical appraisal of clinical utility, challenges, and future research-A consensus document endorsed by the international society of electrocardiology and the international society for holter and noninvasive electrocardiology. Circ Arrhythm Electrophysiol. (2022) 15:e010435. 10.1161/CIR.0000000000001052 35333097 PMC9070127

[11] Bild DE Bluemke DA Burke GL Detrano R Diez Roux AV Folsom AR Multi-ethnic study of atherosclerosis: objectives and design. Am J Epidemiol. (2002) 156:871–81. 10.1093/aje/kwf113 12397006

[12] Friedewald WT Levy RI Fredrickson DS. Estimation of the concentration of low-density lipoprotein cholesterol in plasma, without use of the preparative ultracentrifuge. Clin Chem. (1972) 18:499–502. 10.1093/clinchem/18.6.499 4337382

[13] Whelton PK Carey RM Aronow WS Casey DE Collins KJ Himmelfarb CD 2017 ACC/AHA/AAPA/ABC/ACPM/AGS/APhA/ASH/ASPC/NMA/PCNA guideline for the prevention, detection, evaluation, and management of high blood pressure in adults. J Am Coll Cardiol. (2018) 71:e127–248. 10.1016/j.jacc.2017.11.006 29146535

[14] Levey AS Stevens LA. Estimating GFR using the CKD epidemiology collaboration (CKD-EPI) creatinine equation: more accurate GFR estimates, lower CKD prevalence estimates, and better risk predictions. Am J Kidney Dis. (2010) 55:622–7. 10.1053/j.ajkd.2010.02.337 20338463 PMC2846308

[15] Soliman EZ Alonso A Misialek JR Jain A Watson KE Lloyd-Jones DM Reference ranges of PR duration and P-wave indices in individuals free of cardiovascular disease: the Multi-Ethnic Study of Atherosclerosis (MESA). J Electrocardiol. (2013) 46:702–6. 10.1016/j.jelectrocard.2013.05.006 23806475 PMC3795794

[16] Kamel H Longstreth WT Jr Tirschwell DL Kronmal RA Broderick JP Palesch YY The AtRial cardiopathy and antithrombotic drugs in prevention after cryptogenic stroke randomized trial: rationale and methods. Int J Stroke. (2019) 14:207–14. 10.1177/1747493018799981 30196789 PMC6645380

[17] Hoit BD. Left atrial size and function: role in prognosis. J Am Coll Cardiol. (2014) 63:493–505. 10.1016/j.jacc.2013.10.055 24291276

[18] Hohendanner F Messroghli D Bode D Blaschke F Parwani A Boldt LH Atrial remodelling in heart failure: recent developments and relevance for heart failure with preserved ejection fraction. ESC Heart Fail. (2018) 5:211–21. 10.1002/ehf2.12260 29457877 PMC5880666

[19] Peigh G Shah SJ Patel RB. Left atrial myopathy in atrial fibrillation and heart failure: clinical implications, mechanisms, and therapeutic targets. Curr Heart Fail Rep. (2021) 18:85–98. 10.1007/s11897-021-00510-5 33864224 PMC8994870

[20] Goette A Kalman JM Aguinaga L Akar J Cabrera JA Chen SA EHRA/HRS/APHRS/SOLAECE expert consensus on atrial cardiomyopathies: definition, characterization, and clinical implication. Heart Rhythm. (2017) 14:e3–e40. 10.1016/j.hrthm.2016.05.028 27320515 PMC5548137

[21] Reddy YNV Obokata M Verbrugge FH Lin G Borlaug BA. Atrial dysfunction in patients with heart failure with preserved ejection fraction and atrial fibrillation. J Am Coll Cardiol. (2020) 76:1051–64. 10.1016/j.jacc.2020.07.009 32854840 PMC7455760

[22] Coats AJS Heymans S Farmakis D Anker SD Backs J Bauersachs J Atrial disease and heart failure: the common soil hypothesis proposed by the heart failure association of the European society of cardiology. Eur Heart J. (2022) 43:863–7. 10.1093/eurheartj/ehab834 34875053

[23] Soliman EZ Prineas RJ Case LD Zhang ZM Goff DC Jr. Ethnic distribution of ECG predictors of atrial fibrillation and its impact on understanding the ethnic distribution of ischemic stroke in the atherosclerosis risk in communities (ARIC) study. Stroke. (2009) 40:1204–11. 10.1161/STROKEAHA.108.534735 19213946 PMC2685189

[24] Tiffany WT Ambale VB Volpe GJ Mewton N Rizzi P Sharma RK Associations of electrocardiographic P-wave characteristics with left atrial function, and diffuse left ventricular fibrosis defined by cardiac magnetic resonance: the PRIMERI study. Heart Rhythm. (2015) 12:155–62. 10.1016/j.hrthm.2014.09.044 25267584 PMC4277898

[25] Stalikas N Doundoulakis I Karagiannidis E Kartas A Gavriilaki M Sofidis G Prevalence of markers of atrial cardiomyopathy in embolic stroke of undetermined source: a systematic review. Eur J Intern Med. (2022) 99:38–44. 10.1016/j.ejim.2022.01.024 35065879

[26] Kato Y Takahashi S. Atrial cardiopathy and cryptogenic stroke. Front Neurol. (2022) 13:839398. 10.3389/fneur.2022.839398 35273560 PMC8901724

[27] Gutierrez A Norby FL Maheshwari A Rooney MR Gottesman RF Mosley TH Association of abnormal P-wave indices with dementia and cognitive decline over 25 years: aRIC-NCS (the atherosclerosis risk in communities neurocognitive study). J Am Heart Assoc. (2019) 8:e014553. 10.1161/JAHA.119.014553 31830872 PMC6951047

[28] Liu G Tamura A Torigoe K Kawano Y Shinozaki K Kotoku M Abnormal P-wave terminal force in lead V1 is associated with cardiac death or hospitalization for heart failure in prior myocardial infarction. Heart Vessels. (2013) 28:690–5. 10.1007/s00380-012-0307-9 23160859

[29] Sumita Y Nakatani S Murakami I Taniguchi M. Significance of left atrial overload by electrocardiogram in the assessment of left ventricular diastolic dysfunction. J Echocardiogr. (2020) 18:105–12. 10.1007/s12574-019-00458-5 31813085

[30] Tanoue MT Kjeldsen SE Devereux RB Okin PM. Relationship between abnormal P-wave terminal force in lead V(1) and left ventricular diastolic dysfunction in hypertensive patients: the LIFE study. Blood Press. (2017) 26:94–101. 10.1080/08037051.2016.1215765 27601135

[31] Jin L Weisse AB Hernandez F Jordan T. Significance of electrocardiographic isolated abnormal terminal P-wave force (left atrial abnormality). an echocardiographic and clinical correlation. Arch Intern Med. (1988) 148:1545–9. 10.1001/archinte.1988.00380070053014 2968074

[32] Tsao CW Josephson ME Hauser TH O'Halloran TD Agarwal A Manning WJ Accuracy of electrocardiographic criteria for atrial enlargement: validation with cardiovascular magnetic resonance. J Cardiovasc Magn Reson. (2008) 10:7. 10.1186/1532-429X-10-7 18272008 PMC2244611

[33] Hillis G Moller J Pellikka P Seward J Reeder G Wright R Left atrial volume: a powerful predictor of survival after acute myocardial infarction. Heart. (2003) 107(17):2207–12. 10.1161/01.CIR.0000066318.21784.43 12695291

[34] Overvad TF Nielsen PB Larsen TB Søgaard P. Left atrial size and risk of stroke in patients in sinus rhythm. A systematic review. Thromb Haemost. (2016) 116:206–19. 10.1160/TH15-12-0923 27075168

[35] Lebek S Wester M Pec J Poschenrieder F Tafelmeier M Fisser C Abnormal P-wave terminal force in lead V1 is a marker for atrial electrical dysfunction but not structural remodelling. ESC Heart Failure. (2021) 8:4055–66. 10.1002/ehf2.13488 34196135 PMC8497361

[36] Kamel H Bartz TM Longstreth WT Jr Okin PM Thacker EL Patton KK Association between left atrial abnormality on ECG and vascular brain injury on MRI in the cardiovascular health study. Stroke. (2015) 46:711–6. 10.1161/STROKEAHA.114.007762 25677594 PMC4342300

[37] Tereshchenko LG Shah AJ Li Y Soliman EZ. Electrocardiographic deep terminal negativity of the P wave in V1 and risk of mortality: the national health and nutrition examination survey III. J Cardiovasc Electrophysiol. (2014) 25:1242–8. 10.1111/jce.12453 24837486 PMC4213235

[38] Ahmad MI Singleton MJ Bhave PD Kamel H Soliman EZ. Atrial cardiopathy and stroke mortality in the general population. Int J Stroke. (2020) 15(6):650–6. 10.1177/1747493019876543 31530133

[39] Tereshchenko LG Henrikson CA Sotoodehnia N Arking DE Agarwal SK Siscovick DS Electrocardiographic deep terminal negativity of the P wave in V(1) and risk of sudden cardiac death: the atherosclerosis risk in communities (ARIC) study. J Am Heart Assoc. (2014) 3:e001387. 10.1161/JAHA.114.001387 25416036 PMC4338733

[40] Jacobsson J Carlson J Reitan C Borgquist R Platonov PG. Interatrial block predicts atrial fibrillation and total mortality in patients with cardiac resynchronization therapy. Cardiology. (2020) 145:720–9. 10.1159/000509916 33022672 PMC7677995

[41] O'Neal WT Kamel H Zhang ZM Chen LY Alonso A Soliman EZ. Advanced interatrial block and ischemic stroke: the atherosclerosis risk in communities study. Neurology. (2016) 87:352–6. 10.1212/WNL.0000000000002888 27343071 PMC4977113

[42] Power DA Lampert J Camaj A Bienstock SW Kocovic N Bayes-Genis A Cardiovascular complications of interatrial conduction block: JACC state-of-the-art review. J Am Coll Cardiol. (2022) 79:1199–211. 10.1016/j.jacc.2022.01.030 35331415

[43] Lacalzada-Almeida J Izquierdo-Gómez MM García-Niebla J Elosua R Jiménez-Sosa A Baranchuk A Advanced interatrial block is a surrogate for left atrial strain reduction which predicts atrial fibrillation and stroke. Ann Noninvasive Electrocardiol. (2019) 24:e12632. 10.1111/anec.12632 30719798 PMC6931526

[44] Ariyarajah V Apiyasawat S Najjar H Mercado K Puri P Spodick DH. Frequency of interatrial block in patients with sinus rhythm hospitalized for stroke and comparison to those without interatrial block. Am J Cardiol. (2007) 99:49–52. 10.1016/j.amjcard.2006.07.060 17196461

[45] Chen LY Soliman EZ. P wave indices-advancing our understanding of atrial fibrillation-related cardiovascular outcomes. Front Cardiovasc Med. (2019) 6:53. 10.3389/fcvm.2019.00053 31131284 PMC6509260

[46] Jadidi A Müller-Edenborn B Chen J Keyl C Weber R Allgeier J The duration of the amplified Sinus-P-wave identifies presence of left atrial low voltage substrate and predicts outcome after pulmonary vein isolation in patients with persistent atrial fibrillation. JACC Clin Electrophysiol. (2018) 4:531–43. 10.1016/j.jacep.2017.12.001 30067494

[47] Müller-Edenborn B Minners J Kocher S Chen J Zeh W Lehrmann H Amplified P-wave duration predicts new-onset atrial fibrillation in patients with heart failure with preserved ejection fraction. Clin Res Cardiol. (2020) 109:978–87. 10.1007/s00392-019-01590-z 31863175

[48] Hancock EW Deal BJ Mirvis DM Okin P Kligfield P Gettes LS AHA/ACCF/HRS recommendations for the standardization and interpretation of the electrocardiogram: part V: electrocardiogram changes associated with cardiac chamber hypertrophy: a scientific statement from the American heart association electrocardiography and arrhythmias committee, council on clinical cardiology; the American college of cardiology foundation; and the heart rhythm society: endorsed by the international society for computerized electrocardiology. Circulation. (2009) 119:e251–261. 10.1161/CIRCULATIONAHA.108.191097 19228820

