# Supplementary material for: Electrocardiographic markers of atrial cardiomyopathy and risk of heart failure in the multi-ethnic study of atherosclerosis (MESA) cohort
Source: Front Cardiovasc Med. 2023 Apr 26;10:1143338. doi: 10.3389/fcvm.2023.1143338 (PMC10169752; doi:10.3389/fcvm.2023.1143338)

**Supplemental Material**

**Supplemental Table 1. Association of ECG Markers with Heart Failure in Subgroups**

| **Association of PTFV1 with HF in Subgroups** | | | | |
| --- | --- | --- | --- | --- |
| **Subgroups** | **Events** | **Participants**  **n** | **HR (95% CI)** | **Interaction P-value** |
| **Men** | 34 | 238 | 1.77 (1.20-2.61) | 0.30 |
| **Women** | 16 | 228 | 1.26 (0.73-2.17) |  |
| **Blacks** | 20 | 214 | 1.36 (0.81-2.30) | 0.68 |
| **Non-Blacks** | 30 | 252 | 1.57 (1.06-2.33) |  |
| **≤65 Years** | 18 | 201 | 1.76 (1.02-3.04) | 0.38 |
| **>65 Years** | 32 | 265 | 1.49 (1.02-2.19) |  |
| **Association of aPWA with HF in Subgroups** | | | | |
| **Men** | 28 | 287 | 1.52 (1.01-2.33) | 0.69 |
| **Women** | 18 | 280 | 1.32 (0.79-2.21) |  |
| **Blacks** | 11 | 147 | 1.71 (0.87-3.35) | 0.49 |
| **Non-blacks** | 35 | 420 | 1.37 (0.95-1.99) |  |
| **≤65 Years** | 12 | 256 | 1.48 (0.78-2.78) | 0.37 |
| **>65 Years** | 34 | 311 | 1.39 (0.95-2.03) |  |
| **Association of aIAB with HF in Subgroups** | | | | |
| **Men** | 9 | 37 | 2.37 (1.20-4.67) | 0.71 |
| **Women** | 3 | 19 | 1.84 (0.57-5.90) |  |
| **Blacks** | 2 | 21 | 0.98 (0.23-4.10) | 0.14 |
| **Non-Blacks** | 10 | 35 | 3.00 (1.56-5.76) |  |
| **≤65 Years** | 1 | 11 | 1.72 (0.23-12.7) | 0.47 |
| **>65 Years** | 11 | 45 | 2.49 (1.34-4.61) |  |
| **Association of DTNPV1 with HF in Subgroups** | | | | |
| **Men** | 6 | 32 | 3.06 (1.34-6.99) | 0.28 |
| **Women** | 5 | 25 | 5.33 (2.15-13.2) |  |
| **Blacks** | 5 | 28 | 2.78 (1.11-6.9) | 0.69 |
| **Non-Blacks** | 6 | 29 | 4.06 (1.78-9.24) |  |
| **≤65 Years** | 4 | 22 | 4.22 (1.49-11.9) | 0.59 |
| **>65 Years** | 7 | 35 | 3.69 (1.72-7.91) |  |
| **Association of PWD with HF in Subgroups** | | | | |
| **Men** | 55 | 495 | 1.32 (0.95-1.83) | 0.70 |
| **Women** | 23 | 260 | 1.13 (0.71-1.81) |  |
| **Blacks** | 27 | 267 | 1.41 (0.88-2.27) | 0.93 |
| **Non-Blacks** | 51 | 488 | 1.27 (0.92-1.75) |  |
| **≤65 Years** | 18 | 284 | 1.32 (0.76-2.31) | 0.90 |
| **>65 Years** | 60 | 471 | 1.32 (0.98-1.78) |  |

Abbreviation: PTFV1; p-terminal force in V1; aPWA, abnormal P-wave axis; DTNPV1, deep negativity of p-wave in V1, aIAB, advanced interatrial block; PWD, p-wave duration

Model adjusted for age, sex, race, income, education, smoking status, hypertension, diabetes mellitus, body mass index (continuous), LDL cholesterol, aspirin use, lipid-lowering agents, eGFR and AF.

**Supplemental Figure 1**. ECG tracings of abnormal P-wave indices*. A through **(D)**, Prolonged P-wave duration **(A)**, abnormal P-wave axis **(B)**, abnormal P-wave terminal force in V1 **(C)**, and advanced interatrial block **(D).**


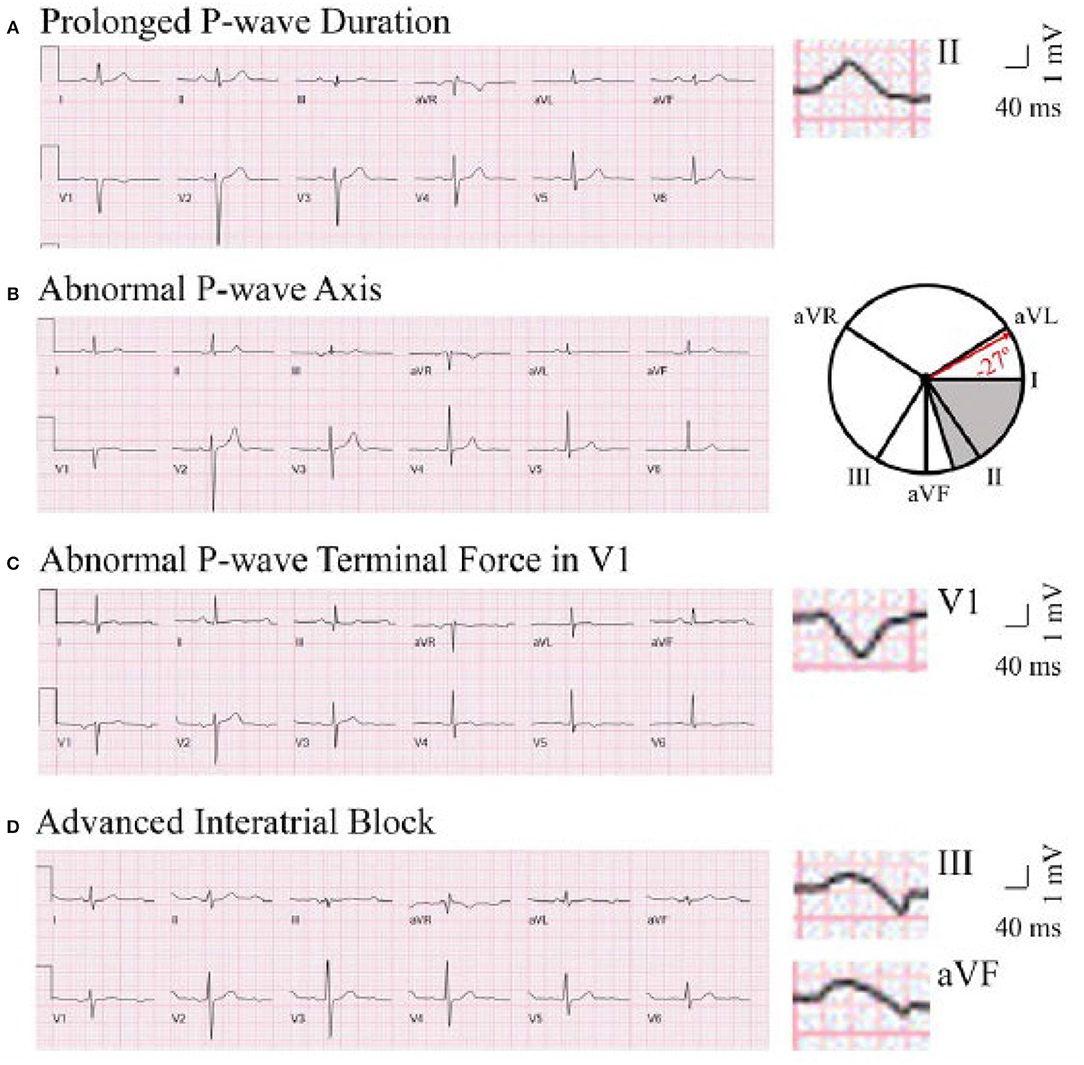


*This figure has been republished from Maheshwari et. al (PMID: 30586710). Drs Chen and Soliman (co-authors) are allowed to publish figure per American Heart Association Policy.

**Supplemental Figure 2. Adjusted Kaplan Meier Curves for all Incident Heart Failure Stratified by Each ECG Predictor**


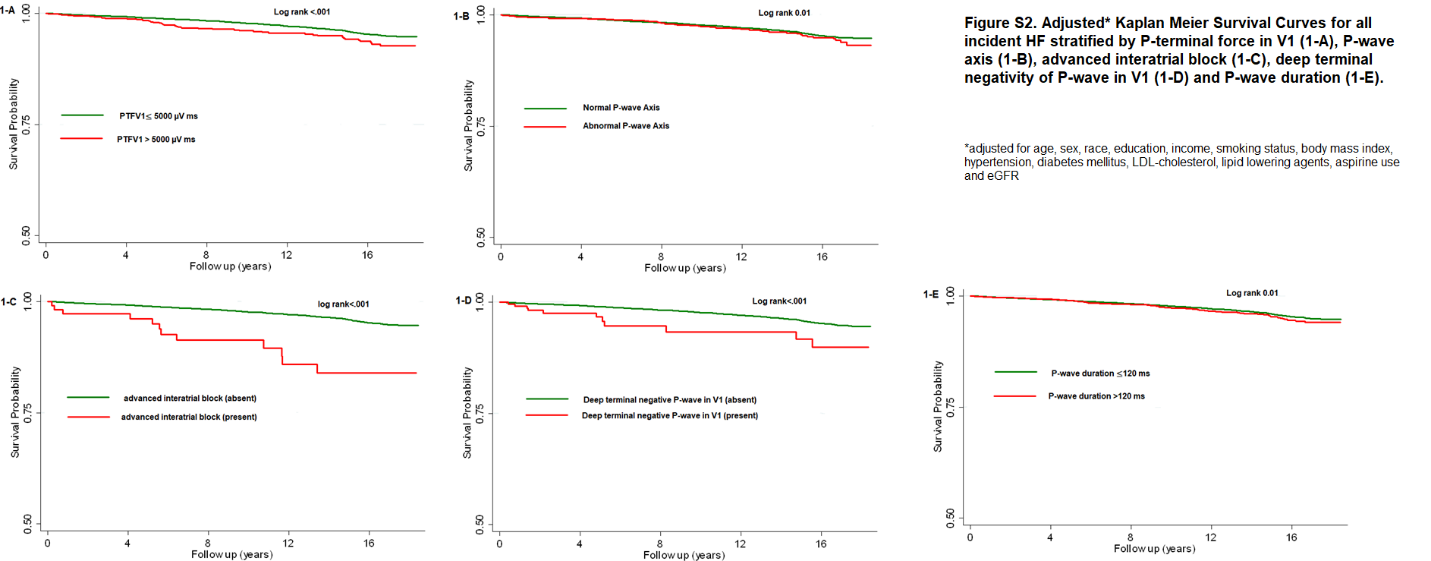

Supplement: Supplementary file 1 [file Table1.docx]
